# Supplementary material for: 1H-NMR Approach for the Discrimination of PDO Grana Padano Cheese from Non-PDO Cheeses
Source: Foods. 2024 Jan 23;13(3):358. doi: 10.3390/foods13030358 (PMC10855172; doi:10.3390/foods13030358)
Supplement: Supplementary file 1 [file foods-13-00358-s001.zip › foods-2788427-supplementary.pdf]

^{1}H-NMR Approach for the Discrimination of PDO Grana Padano Cheese from Non-PDO Cheeses

SUPPLEMENTARY MATERIALS

Valentina Maestrello[1,2], Pavel Solovyev[1], Pietro Franceschi[1], Angelo Stroppa[3] and Luana Bontempo[1]*

[1]Fondazione Edmund Mach (FEM), Centre for Research and Innovation (CRI), Via E. Mach 1,
38098 San Michele all'Adige, TN, Italy; valentina.maestrello@unitn.it (V.M.); pietro.franceschi@fmach.it (P.F.);
luana.bontempo@fmach.it (L.B.)

[2]Centre for Agriculture, Food and Environment (C3A), University of Trento, Via E. Mach 1,
38098 San Michele all'Adige, TN, Italy

[3]Consorzio Tutela Grana Padano, Via XXIV Giugno 8, San Martino Della Battaglia,
25010 Desenzano del Garda, BS, Italy; a.stroppa@granapadano.com

* Correspondence: pavel.solovyev@fmach.it; Tel.: +39-0461-615627

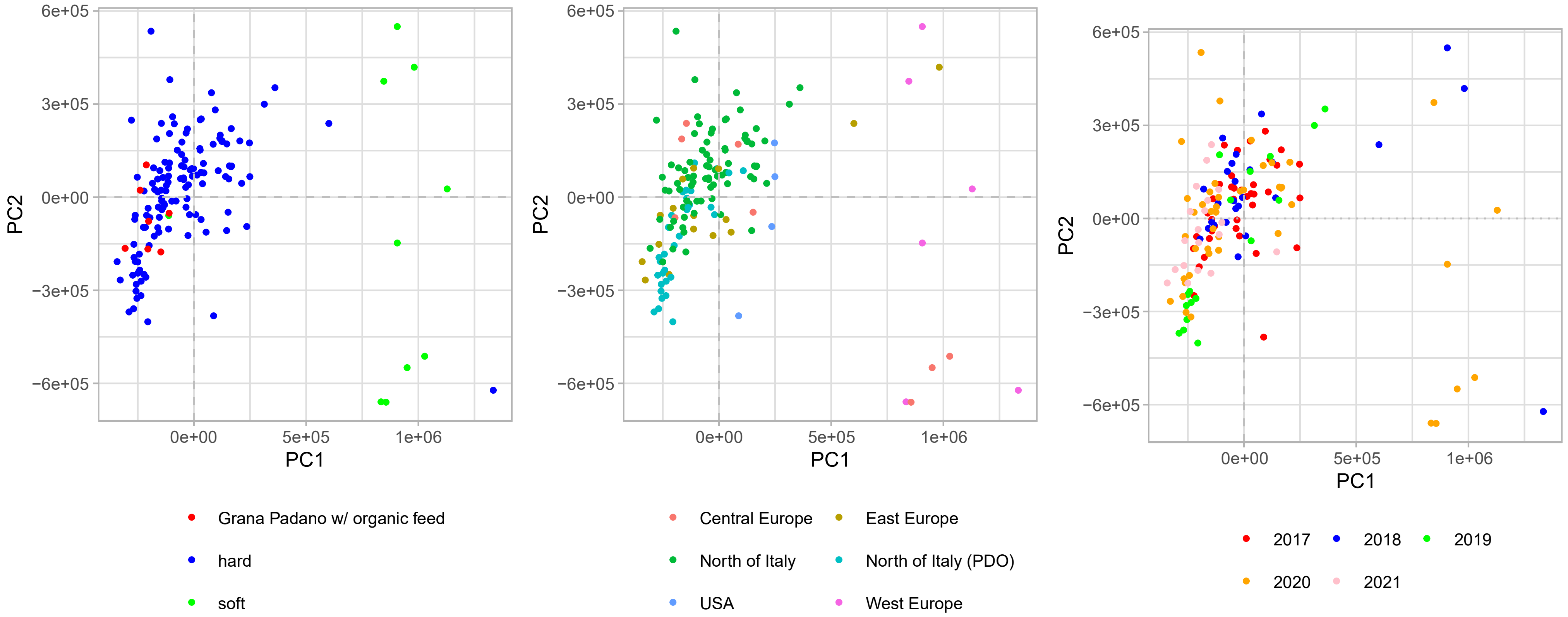

Figure S1: PCA plots of aqueous fraction with different colors of the dots to highlight relevant metadata: the type of cheese (hard cheeses, including Grana Padano, soft cheeses and Grana Padano defined as organic because of the different feed used), the area of production and year of production

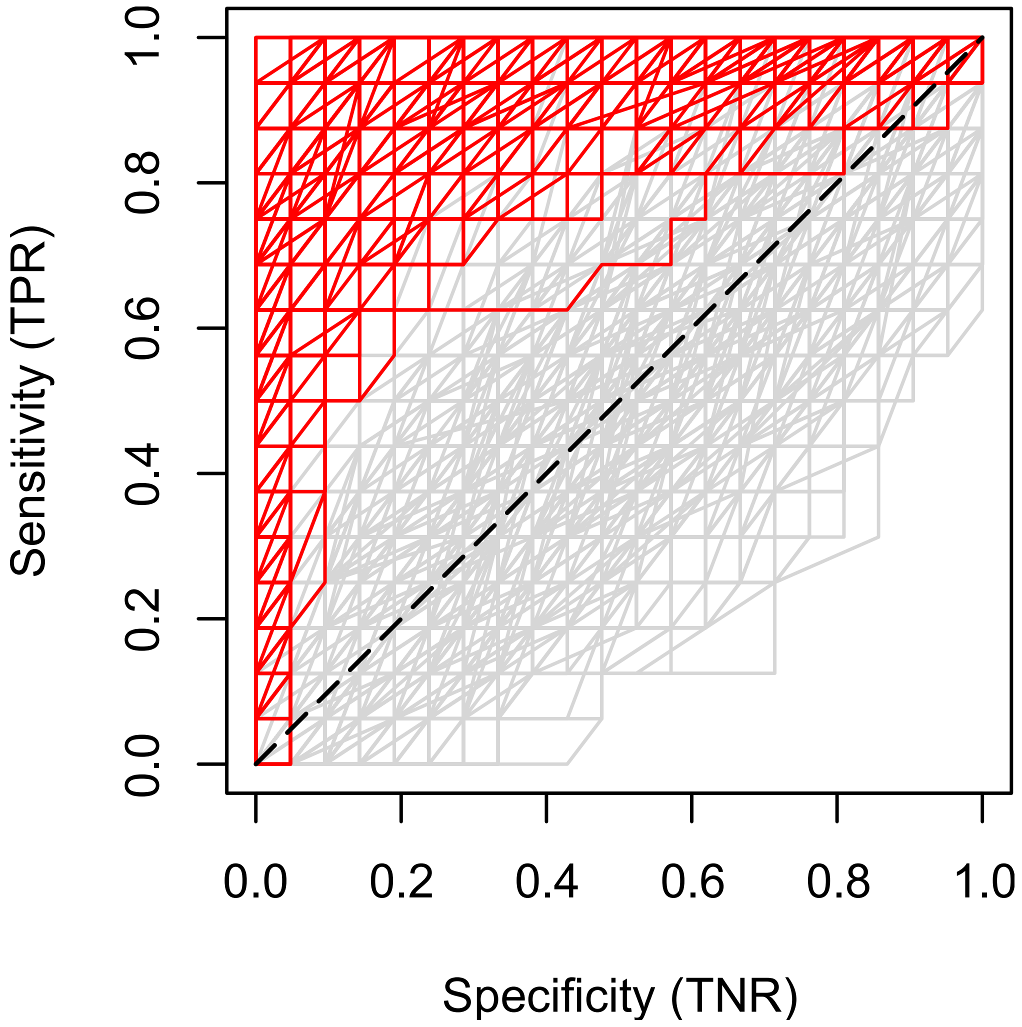

Figure S2: ROC curves for 500 RF models (in red) and ROC curves for 500 RF models with random labels (in gray) for the aqueous extract

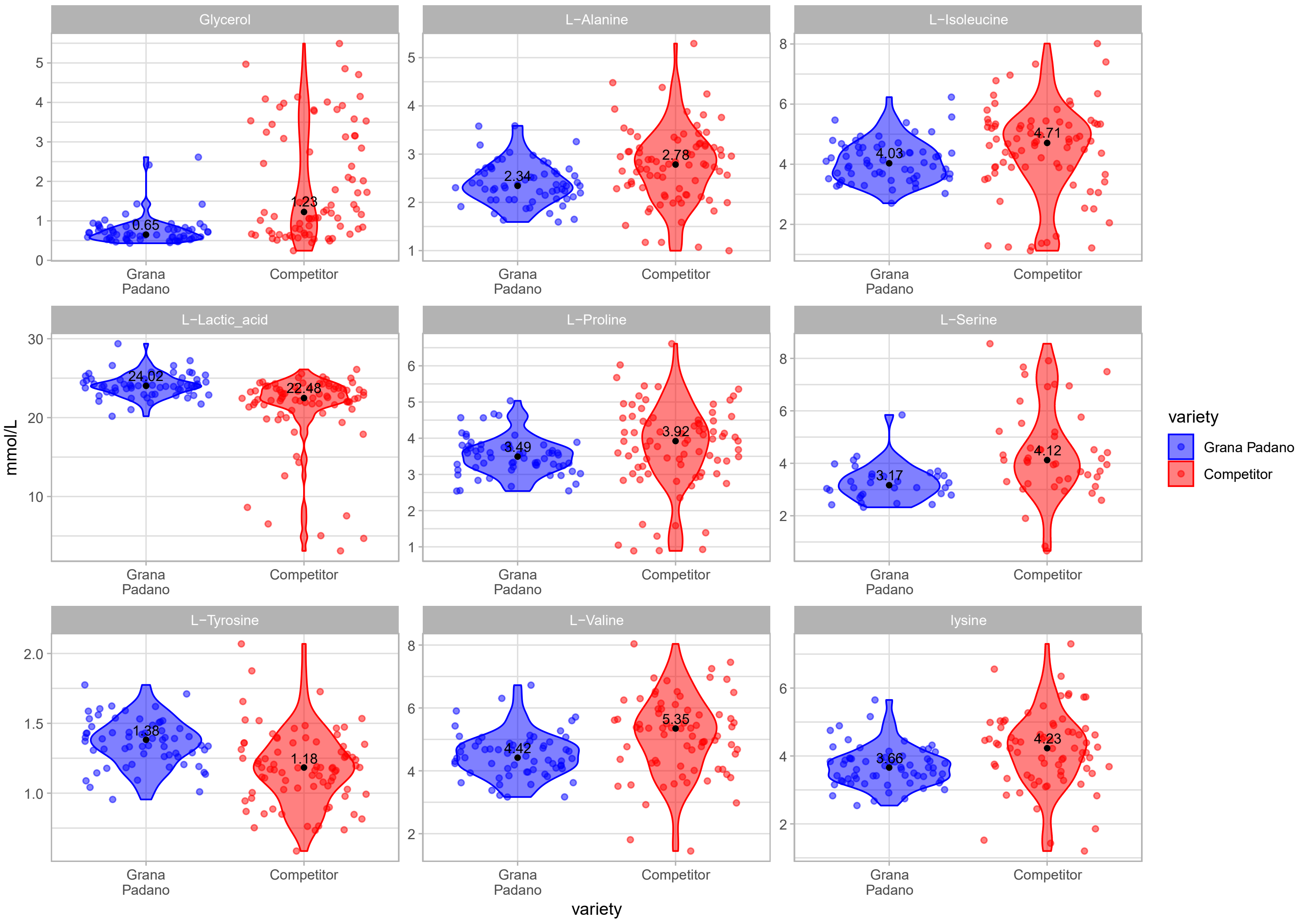

Figure S3: Violin plots of the most discriminant compounds quantified with AssureNMR (results are reported in mmol/L and corrected by the weight). The black dot shows the median of the values.

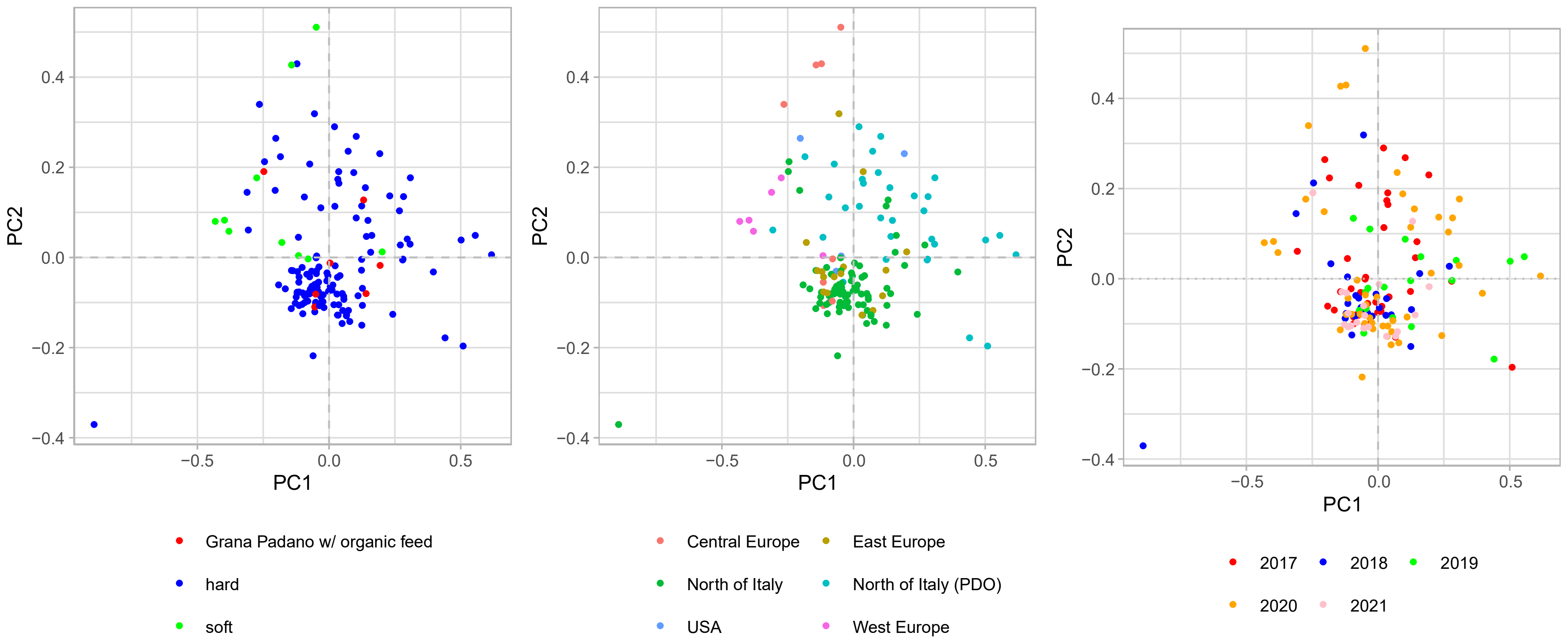

Figure S4: PCA plots of lipid fraction with different colors of the dots to highlight the relevant metadata: the type of cheese (hard cheeses, including Grana Padano, soft cheeses and Grana Padano defined as organic because of the different feed used), the area of production and year of production.

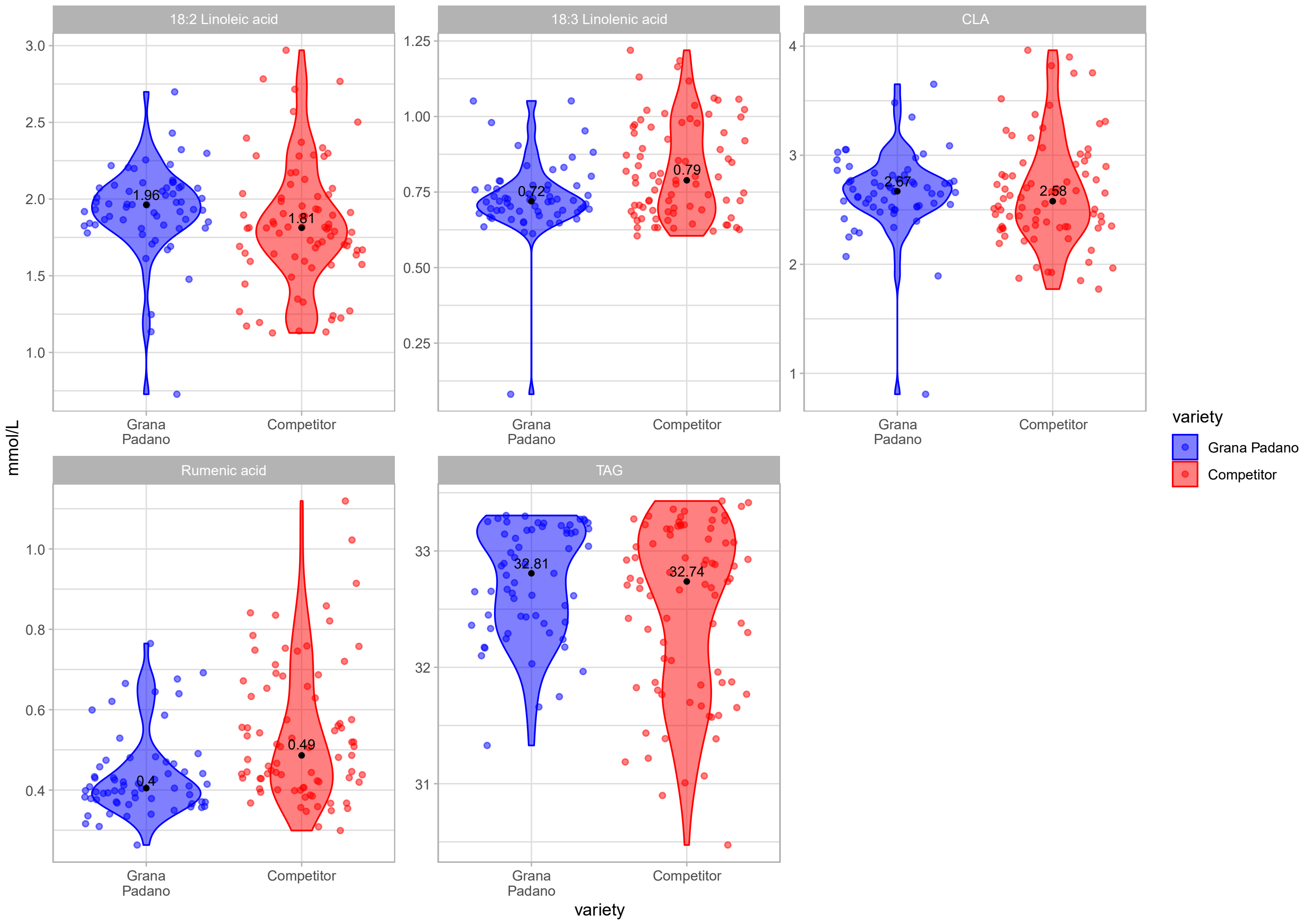

Figure S5: Violin plots of the most discriminant compounds quantified with AssureNMR (results are reported in % molar fraction). The black dot shows the median of the values.

PLS-DA

Since PLS-DA is one of the most adopted multivariate approaches, it was applied to this dataset to assess its discriminating potential with such a diverse dataset. The model was built with the mdatools package [1] on the same dataset and with the same pre-treatments as for Random Forest model. In brief, the dataset was split into training and test sets (80:20) and the model complexity (number of PLS components) was optimized on the training set with a leave-one-out scheme. The score plots of both the fractions are shown in Figure S6. Looking at Figure S6, it is evident that the PLS model did not clearly separate the two groups neither in the aqueous nor in the lipid fractions. In terms of performance on the test set, the model showed a rather high misclassification rate: 23% for the aqueous model (7 out of 30) and 20% for the lipid model (6 out of 30). These results support the choice of relying on a more flexible RandomForest approach for the construction of the final model.

1. Kucheryavskiy S. Projection based methods for preprocessing, exploring and analysis of multivariate data used in chemometrics. **2020**. https://doi.org/10.1016/j.chemolab.2020.103937

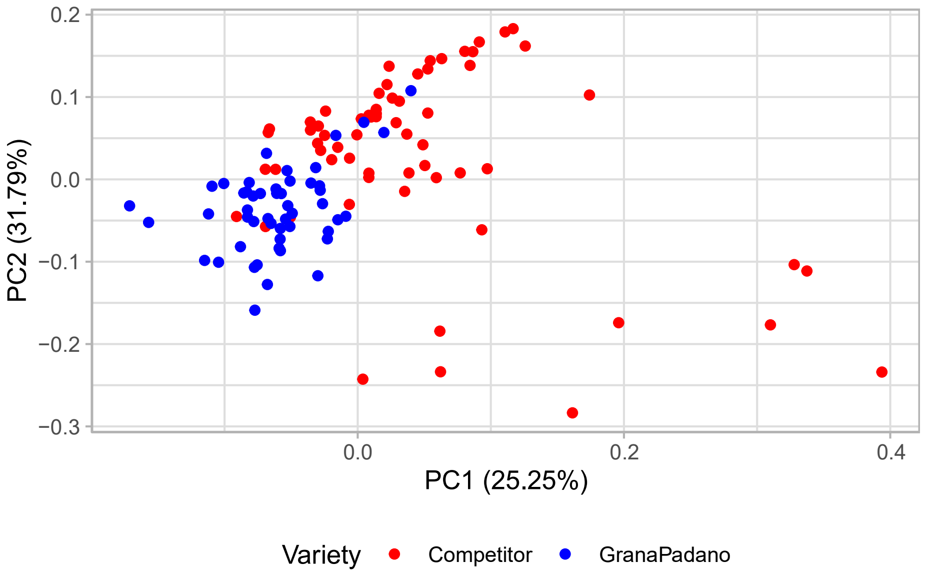

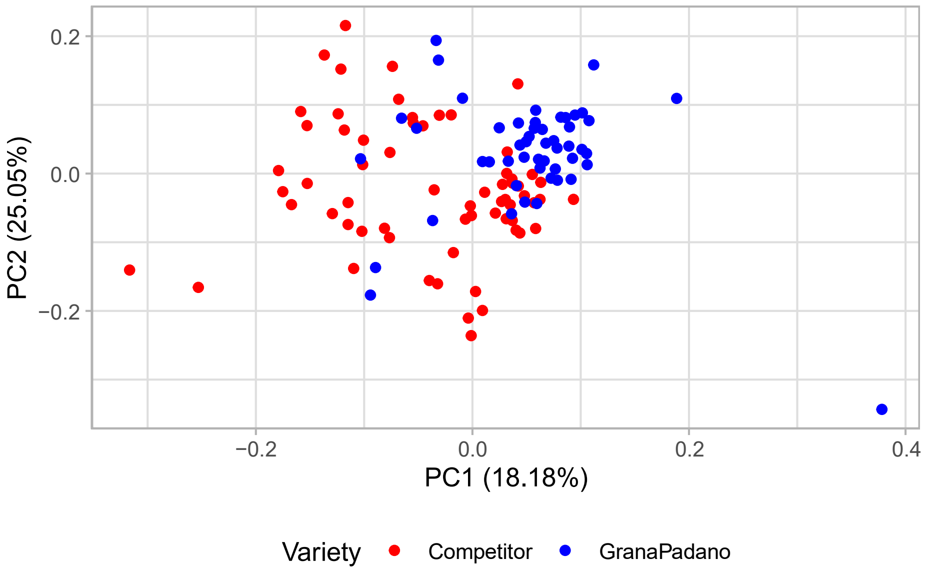

Figure S6: PLS-DA score plots for aqueous fraction (above) and lipid fraction (below). The dots are colored according to their class.
